# The use of long-acting injectable antipsychotics in an acute psychiatric unit

**DOI:** 10.1192/j.eurpsy.2023.438

**Published:** 2023-07-19

**Authors:** G. Ortega-Hernández, N. Ramiro, F. Palma-Álvarez, Ó. Soto-Angona, M. F. Mantilla, J. Duque, I. Gonzalo, F. Collazos

**Affiliations:** ^1^Hospital Vall d´Hebrón; ^2^Hospital San Rafael; ^3^Hermanas Hospitalarias-CSMIA Gracia, Barcelona, Spain

## Abstract

**Introduction:**

Long-acting injectable antipsychotic (LAI) are an important and arguably under-utilized therapeutic option, particularly where medication adherence is a priority (Pilon et al. Clin Ther 2017; 39 1972-1985).

In recent years, meta-analytic reviews of depot medications concluded that this route of administration produced clinical advantages in terms of overall outcome, with lower probability of relapse, readmissions, shorter hospital admission time, mortality, and thus better long- term prognosis over other oral antipsychotics (Leucht et al. Schizophr Res 2011;127 83-92). Depot treatment is associated with lower overall medical expenditure (Taipal et al. Schizophr Bull 2018;17 1381- 1387).

**Objectives:**

To describe the evolution of people diagnosed with a psychotic disorder 6 months before and after the introduction of long-acting injectable antipsychotic (LAI) in the acute psychiatric unit of San Rafael Hospital (Spain) from January 1, 2018 to December 31, 2018.

**Methods:**

Retrospective and prospective naturalistic study. Patients with a diagnosis of psychotic disorder who were admitted to the acute psychiatric unit in 2018 and who were introduced to LAI (paliperidone palmitate, aripiprazole, olanzapine pamoate or risperidone), are selected. Sociodemographic variables (sex, age, ethnicity, migratory status, marital status, occupation, cohabitation) and clinical variables (main and secondary diagnosis, comorbidity with drug use and history of poor adherence) are described. The number of emergency visits and hospital admissions before and after the introduction of LAI antipsychotic treatment is compared.

**Results:**

The sample was composed of 99 subjects. The mean age was 42.46 years (SD 13.439) and 67.7% were men. The socio-demographic profile was: european caucasian ethnicity (73.7%), non- migrant status (69.7%), single (67.7%), inactive (43.4%) and residing in the home of relatives (50.5%). 53.5% have a diagnosis of schizophrenia, followed by schizoaffective disorder (24.2%). 45.5% are diagnosed with any drug use disorder, the most frequent being cannabis (30.3%). 76.8% have a history of discontinuing oral treatment. There was a statistically significant decrease (p<0.0001) in number of emergency visits and hospital admissions after the introduction of LAI antipsychotic.

In the general linear multivariate before-after model, there were significant differences (p=0.002) in the number of admissions after long-term IM antipsychotic treatment. As for the comparison of the effects between the different LAIs, there are differences between them (p< 0.0001). Post-hoc analysis (Bonferroni) only showed differential significance for treatment with Paliperidone Palmitate (p<0.0001).

**Conclusions:**

The use of LAI antipsychotic can reduce the number of emergency room visits and hospital admissions, in line with literature.

**Disclosure of Interest:**

None Declared

